# The effect of climate change on cholera disease: The road ahead using artificial neural network

**DOI:** 10.1371/journal.pone.0224813

**Published:** 2019-11-06

**Authors:** Zahra Asadgol, Hamed Mohammadi, Majid Kermani, Alireza Badirzadeh, Mitra Gholami

**Affiliations:** 1 Department of Environmental Health Engineering, School of Public Health, Iran University of Medical Sciences, Tehran, Iran; 2 Department of Environmental Health Engineering, School of Public Health, Zanjan University of Medical Sciences, Zanjan, Iran; 3 Research Center for Environmental Health Technology, Iran University of Medical Sciences, Tehran, Iran; 4 Department of Parasitology and Mycology, School of Medicine, Iran University of Medical Sciences, Tehran, Iran; Jawaharlal Nehru University, INDIA

## Abstract

Climate change has been described to raise outbreaks of water-born infectious diseases and increases public health concerns. This study aimed at finding out these impacts on cholera infections by using Artificial Neural Networks (ANNs) from 2021 to 2050. Daily data for cholera infection cases in Qom city, which is located in the center of Iran, were analyzed from 1998 to 2016. To determine the best lag time and combination of inputs, Gamma Test (GT) was applied. General circulation model outputs were utilized to project future climate pattern under two scenarios of Representative Concentration Pathway (RCP2.6 and RCP8.5). Statistical downscaling was done to produce high-resolution synthetic time series weather dataset. ANNs were applied for simulating the impact of climate change on cholera. The observed climate variables including maximum and minimum temperatures and precipitation were tagged as predictors in ANNs. Cholera cases were considered as the target outcome variable. Projected future (2020–2050) climate in previous step was carried out to assess future cholera incidence. A seasonal trend in cholera infection was seen. Our results elucidated that the best lag time was 21 days. According to the results of downscaling tool, future climate in the study area by 2050 will be warmer and wetter. Simulation of cholera cases indicated that there is a clear trend of increasing cholera cases under the worst scenario (RCP8.5) by the year 2050 and the highest cholera cases observe in warmer months. The precipitation was recognized as the most effective input variable by sensitivity analysis. We observed a significant correlation between low precipitation and cholera infection. There is a strong evidence to show that cholera disease is correlated with environment variables, as low precipitation and high temperatures in warmer months could provide the swifter bacterial replication. These conditions in Iran, especially in the central parts, may raise the cholera infection rates. Furthermore, ANNs is an executive tool to simulate the impact of climate change on cholera to estimate the future trend of cholera incidence for adopting protective measures in endemic areas.

## Introduction

Studies have elucidated the effects of climate change on infectious diseases, including water, vector and food-borne diseases [1–3]. There is the evidence of relationship between climatic parameters and the burden of water-borne diseases such as cholera, particularly in undeveloped/developing countries [4–6].

The cause of cholera is *Vibrio cholerae* (*V*. *cholerae*), which is categorized as a gram-negative bacterium. Its natural habitat is brackish water. The disease is a severely contagious acute bacterial infection, which is caused by colonization and multiplication of *V*. *cholerae* inside the intestine. High risk individuals get the infection when they ingest an infective dosage of bacteria from contaminated water, vegetables and foods [7]. According to the World Health Organization (WHO) reports, cholera is one of the health threats in some developing countries. In 2017, a total of 1227391 cases of cholera were reported by 34 countries including 5654 deaths [8]. Cholera infection has emerged in association with seasonality, travel, natural catastrophes, warfare and distinct conditions which ends to insufficient sanitation and poverty [9]. With growing concerns about global climate change, the role of climatic factors in cholera incidence has been investigated in the last decades. Weather conditions such as increasing ambient temperature were known as a key parameter for cholera incidence [10, 11]. Several studies have shown a significant correlation between either high or low rainfall and incidence of cholera cases. So, either increasing or decreasing the average rainfall could lead to flood and droughts which can affect the concentration of bacteria and also human health [12–14]. Rainfall can also effect on nutrient concentrations, salinity and pH of water resources which effect on bacterial survival [15].

General Circulation Models (GCMs) have been applied to evaluate the effect of climate change on a wide region. The Intergovernmental Panel on Climate Change (IPCC) has published the latest sets of scenarios (Representative Concentration Pathway (RCP)) in the its fifth Assessment Report (AR5) in 2014. These scenarios include RCP8.5, RCP6, RCP4.5, and RCP2.6 [16]. To link between outputs of GCMs (spatial resolution of 100–300 km) and the local climatic processes (spatial resolution of 10–20 km), downscaling technique was investigated. This technique was applied to project future climate pattern at the local scale under RCP scenarios [17].

Recently, the prediction of effect of climate change on health aspects has been widely studied [18, 19]. Prediction of an infectious disease such as cholera by reliable modeling can help managers to perform preventive actions such as preparation of adequate manpower, pharmaceutical and logistic resources [20]. Artificial neural networks (ANNs), as nonlinear statistical modeling, can be applied for creating models of disease spreading and prediction of epidemic outcomes. Moreover, ANNs can gain elucidation of data and nonlinear forecast analysis of variables for evaluation of biological and environmental data [21]. Several studies applied ANNs to forecast the incidence of cholera over an area and illustrated advantages and disadvantages of this method [21, 22].

Many undeveloped/developing countries such as Iran still suffer from frequent epidemics; therefore, the objectives of present study were evaluation of the association between the cholera cases and climate variables and also finding the most effective parameters. In addition, we developed ANNs to simulate the climate change impact on cholera cases by the year 2050 in Qom, Iran, with the highest cholera incidences, within two scenarios of RCP2.6 and RCP8.5. In considering importance of controlling cholera in Qom, it can help to develop a system for warning incidence of cholera which lead to promotion of planning and decision-making for public health and reducing the health effects of climate change.

## Material and methods

### Study area

**Fig 1:**
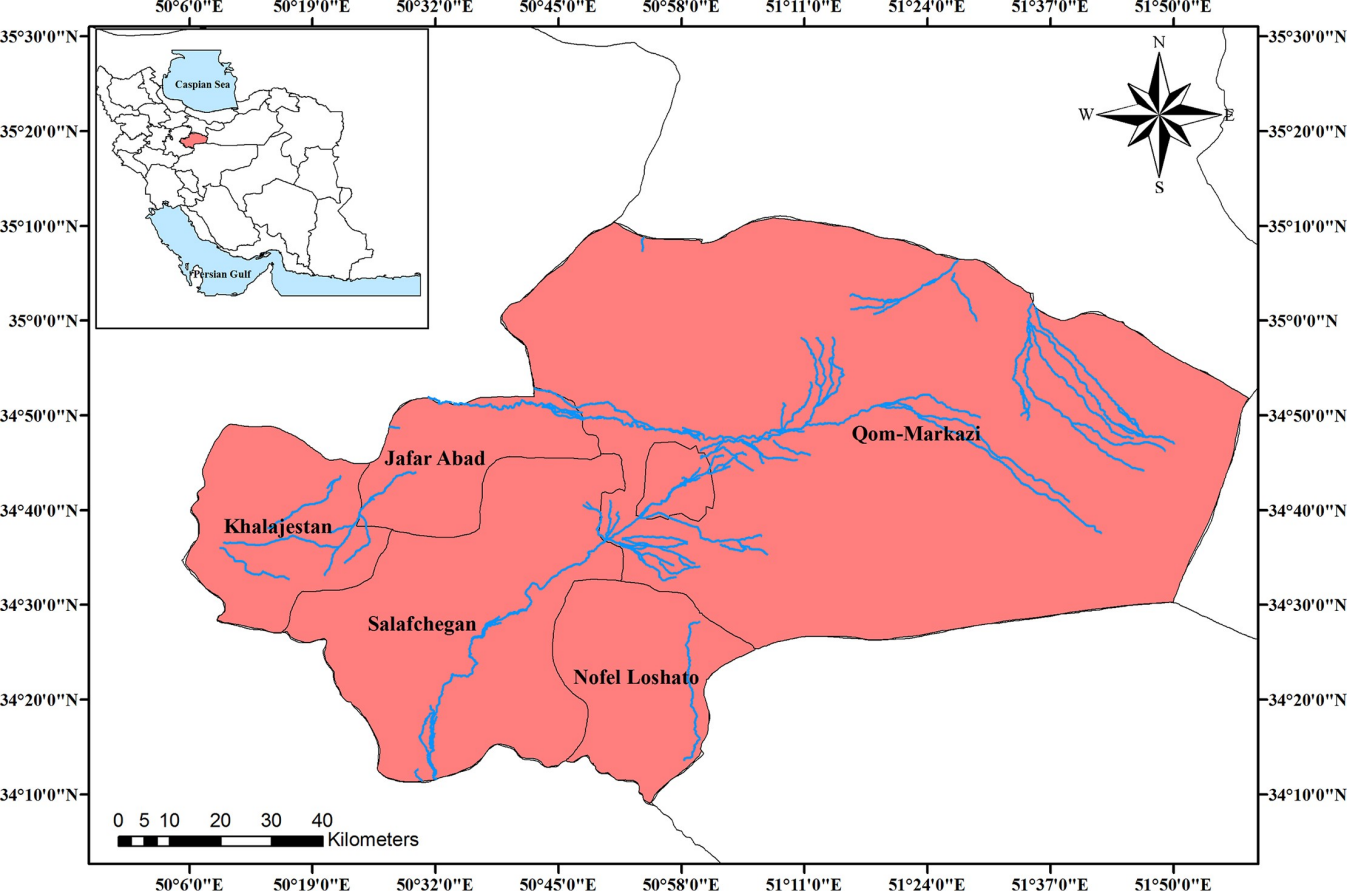
Study area: Qom, Iran (created by Arc GIS version 10.2).

The study area was Qom (50.88°N 34.64°E), located in the center of Iran and southwest of Tehran (capital of Iran) (Fig 1). Qom is one of the biggest, industrialized and densely populated cities in Iran, with an urban population of over 1 million. The region’s physical geography is dry and warm with an average annual maximum temperature of 40.3°C in July and the average annual minimum temperature of -1.6°C in January. Annual rainfall average is 125 mm which occurs between October and May [23, 24].

### Data source and collection

#### Cholera

Daily cholera prevalence data from January 1998 to December 2016 were collected from the records of "The Centers for Disease Control and Prevention" at Qom University of Medical Sciences. Systematic cholera surveillance has been started working from 1998 epidemic in Qom, Iran; therefore, there was not available any cholera data for previous year. The suspected cholera cases were diagnosed by the conventional culture technique and confirmed as cholera case-counts.

#### Meteorological data

Meteorological data were collected from Iran Meteorological Organization (IMO) for the period of 1976 to 2016. Data were collected regarding daily minimum temperatures (T_min_), daily maximum temperatures (T_max_) (in °C) and daily precipitation (pr) (in mm) from Qom meteorological stations.

### Preprocessing and optimization processes

The Gamma Test (GT) was known as a non-linear modeling analysis tool which estimated the minimum Mean Square Error (MSE). GT can be attained when modeling the unseen data using any continuous nonlinear models. This tool was firstly introduced by Koncar [25] and Agalbjörn [26] and then improved by other researchers [27, 28]. GT is an index of strength association between two variables measured at the ordinal level. This index estimated that part of variance of output data which cannot be accounted for any smooth data model and calculate by vertical intercept of the regression line [29]. Furthermore, slope or gradient is the slope of regression line and could provide information on complexity of the system. GT is known as a non-parametric method and its results used to build a model regardless of the techniques. The result can standardize by V-ratio, which determines as Gamma/Var (output) and scale between 0 and 1. A V-ratio equal or close to zero demonstrates a high degree of predictability of given output. Furthermore, if the standard error (SE) value tends to zero, there is a high confidence in the value of gamma statistic as an estimate for the noise variance on the output [30]. To estimate the reliability of gamma statistic, a series of GT (for a definite number of unique data points (M)) was run to determine the size of the data set required to create a stable asymptote. The M-test helps us to find out how many data points are adequate for building a model with a proximate MSE to the estimated noise variance (when the M-test plot be-comes flat) [31].

In the present study, different combinations of input data were explored to assess their influence on the cholera disease. GT evaluated the best combination of the candidate inputs by Genetic Algorothim (GA). The parameters of GA to find good embedding were the population size (100 chromosomes), the mutation rate (0.01), the crossover rate (0.5), the gradient fitness (0.1), intercept fitness (0.8), and length fitness (0.1) [16]. The non-linear analysis and modeling tool which used to analyze the data was winGamma that developed by the Department of Computer Science, Cardiff University [29].

In the first step, five time-series data including cholera's data with no lag time (NL), cholera's data with seven days lag (7DL), cholera's data with 14 days lag (14DL), cholera's data with 21 days lag (21DL) and cholera's data with 30 days lag (30DL) were prepared to be examined. In the next step, standard normalization was applied to give an equal chance to contribute to an output prediction for each data group. Then, different smooth data modeling techniques were built and examined. Three principal factors of GT including gamma statistics, model complexity, and the required number of inputs were used to determine the fitness of particular mask or feature set. Finally, according to the results of GT, GA and M-test, the best lag time was chosen between five time-series.

### Projecting future climate patterns and climate scenario

Simulated climate change data on monthly scales in baseline experiments (1976 to 2005) and future experiments (2021 to 2050) were obtained from Intergovernmental Panel on Climate Change (IPCC)-data distribution center (DDC) website. The future experiments were included four RCP scenarios (RCP8.5, RCP6, RCP4.5, and RCP2.6), which in the present study, we used two of the RCP scenarios, RCP8.5 and RCP2.6 (with a relatively high and low future pathway). In this study, ten global climate change model outputs (France IPSL-CM5A-LR, Japan MIROC5, Canada CanESM2, USA CESM1-CAM5, Australia CSIRO-Mk3-6-0, China FIO-ESM, South Korea HadGEM2-AO, UK HadGEM2-ES, Germany MPI-ESM-MR, China bcc-csm1-1) were applied to project the future climate. ArcGIS10.2 was utilized to display and transfer Net-CDF (network common data form) files to excel. Monthly average of all variables (baseline and future) were calculated and graphed separately. Then, the above-mentioned data of the two selected groups in 10 models were compared. Furthermore, change factor method was used to select the most appropriate GCMs.

Statistical downscaling establishes the relationships between the selected GCM outputs and observed regional climatic processes [32]. LARS-WG (Long Ashton Research Station Weather Generator, version 5–5) was applied as a successful downscaling tool, to both generate or simulated future daily meteorological variables in our study area [33]. The process of creating daily site-specific climate data were carried out in three steps as follows:

**1. Site analysis (model calibration):** In this step, observed daily weather data from 1976 to 2005 (30 years) recorded in Qom synoptic stations was first used to calculate their statistical parameters. These parameters were applied by LARS-WG in next step to generate synthetic data series. The Kolmogorov-Smirnov (K-S) were used to determine the best copula fitting to data, t-test for monthly means, and F-test for standard deviation [34].

**2. Model validation:** Synthetic weather series (generated by the WG) were methodologically tested against observed data to find out the presence of any statistically significant difference in the statistical parameters. This method led to increasing the confidence in the models’ predictions [33].

**3. Generation of future daily synthetic weather data:** LARS-WG baseline parameters which are derived from observed weather data (during the process of model calibration) are applied to generate synthetic weather by using the output of GCMs (as predictors). It derived changes in precipitation and temperature and statistically downscale the conditioning of parameters. The LARS-WG version of 5–5 has not covered the latest sets of scenarios which released in the IPCC-AR5. Thus, to produce the RCP scenario fills and generate future weather data, the observed climate data in the study area was compared with the GCM outputs and then applied to the LARS scenario fill.

### Simulation

The ANNs were applied to simulate the climate change impacts on cholera disease. ANNs is a powerful mechanism and an important computational data-driven model which can learn a correlation between inputs and outputs (training) and then represents the relationship between input and output parameters by building a model. One of the most commonly and effective used types of neural network is Back Propagation Neural Network (BPNN) approach, i.e. Multilayer Perceptron (MLP). Using MLP, a learning model created by baseline data and then it uses to produce outputs for new inputs [35].

In the present study, the software package Neuro-Solutions for Excel, version 7 (Neuro-Dimension, Inc. 3701 NW 40th Terrace, Suite 1 Gainesville, FL 32606, NeuroSolutions for Excel) was applied. The observed climate datasets in the study area were tagged as inputs and the number of cholera disease was tagged as desired in an ANNs model for training, cross-validation, and testing. MLPs algorithm was used to create a neural network model and predict cholera disease number, due to climate change. MLPs are structured of three layers of input, hidden, and output and two steps of input feed-forward network and back propagation. After randomization of the datasets, for training, cross-validation, and testing the network, 60, 15 and 25% of the inputs and outputs datasets were applied, respectively. In this study, to evaluate the prediction success, we assessed the accuracy of the estimate by using MSE, correlation coefficients (R-values), root mean square error (RMSE), normalized mean square error (NMESE), and mean absolute error (MAE). Finally, the future (2021–2050) trends of cholera disease were estimated under two RCP scenarios (RCP2.6 and RCP8.5) using the projected climate datasets in the optimized ANNs.

### Statistical analysis

For statistical analysis of collected data, SPSS software (version 20) was applied and the normality of all data was evaluated by Kolmogorov-Smirnov test. Descriptive statistics were used for analysis of meteorological data and the number of cholera cases. Furthermore, the relationship between the number of cholera cases and climate variables was determined by Pearson correlation coefficient. For all tests, *P*-values of ≤0.05 are embraced as statistically significant. GraphPad Prism 8.0.1 was used to design and graph in the current study. Also, ArcMap 10.2 Geographical Information System (GIS) (ESRI, Redlands, CA) was applied as a suitable tool for mapping the study area and transferring Net-CDF (network common data form) files to excel.

## Results

### Overview of effect of climate variability on cholera

**Fig 2:**
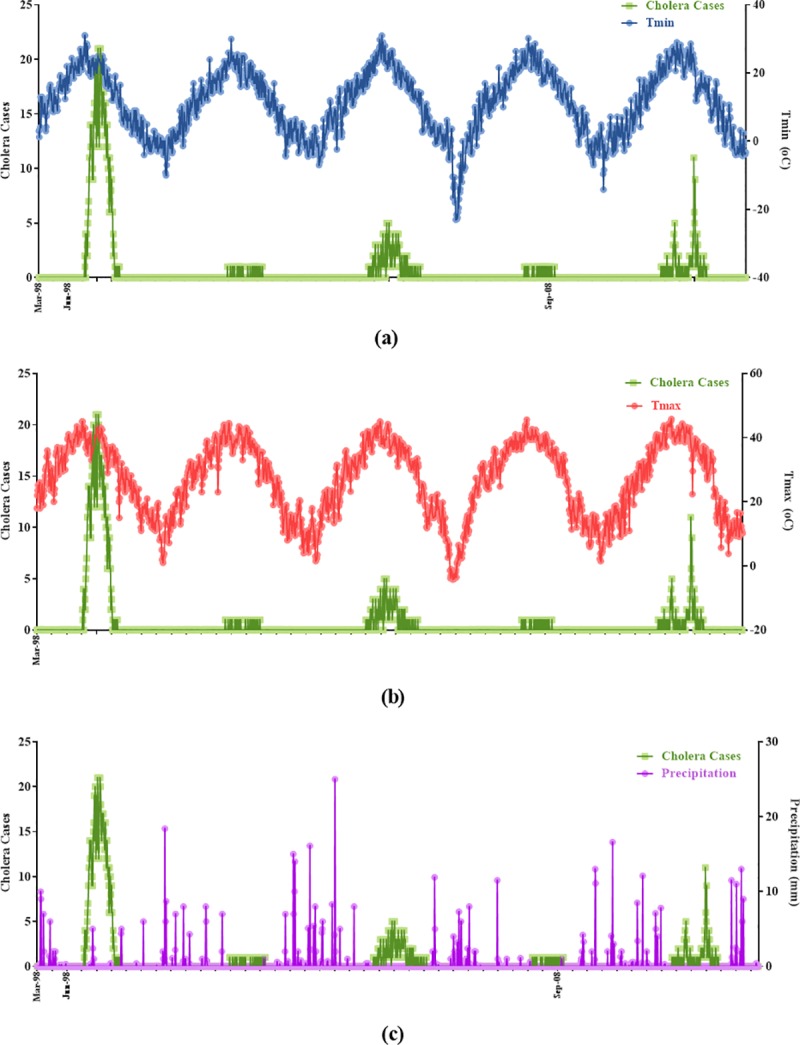
Variation in the number of cholera cases and climate variables in five years (1998, 2001, 2005, 2008 and 2011).

**Fig 3:**
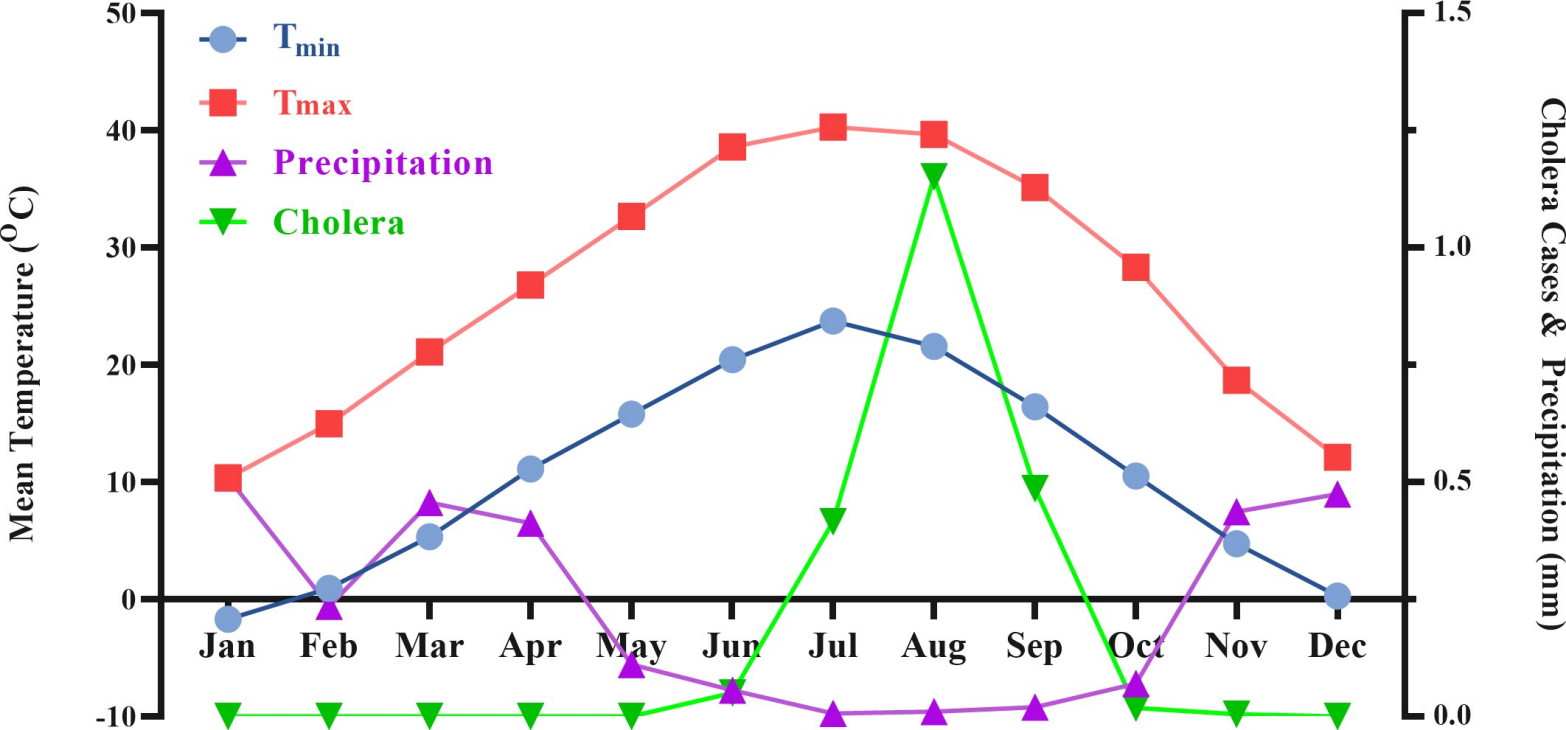
Monthly variation in the number of cholera cases and climate variables, 1998–2016.

From March 1998 to December 2016, in total, 1243 patients were identified with cholera by the surveillance system in Qom province. The highest number of annual cholera cases were recognized in five years 1998, 2001, 2005, 2008 and 2011 with 836, 22, 167, 26 and 137, respectively, and the other years have few or zero number of cholera cases. The minimum, maximum and mean of monthly variables for each year are presented in Table 1. The maximum monthly of cholera was 21 in August 1998, as a year with the highest number of cholera cases. No case was recognized in some years observed in Table 1. The monthly average of minimum temperature varied between 10.5°C in 2000 and 11.54°C in 2015 which shows an increase for minimum temperature. The lowest temperature was recorded in January 2008 with a monthly average of -23°C after a catastrophic weather in Qom. An increasing trend was observed for monthly average of maximum temperature. So, the maximum temperature was elevated from 43.5°C in August 1998 to 45.2°C in June 2016 and also during 19 years, the highest temperature was recorded in July 2010 with 47°C. The maximum precipitation was varied between 9.5 mm in December 2014 and 35 mm in November 1999. Furthermore, a decreasing trend was observed for precipitation during the years. Time series of cholera and climate variables were applied to demonstrate the relationship between climatic factors and number of cholera cases (Fig 2). For better understanding the figure, we presented the monthly values of cholera cases and climate variables in just 5 years with the highest number of choleras and ignored the years with negligible or zero cases of cholera. A seasonal trend in cholera cases and climate variables is shown during 1998 to 2016 (Fig 3). Highest numbers of cholera were observed during the warm season from June to September with highest maximum and minimum temperature and lowest precipitation. Analysis of the correlation between monthly average of climate variables and number of cholera cases were carried out by Pearson test (Table 2). The maximum and minimum temperatures as independent variables had a positive correlation and also precipitation has a negative correlation with number of cholera cases (R^2^ = 0.211, R^2^ = 0.204 and R^2^ = -0.226, respectively). Moreover, the seasonal pattern of cholera disease was confirmed by correlation coefficient of 0.332 between months and the number of cholera cases.

**Table 1:** Descriptive statistics of the monthly climatic variables and cholera cases.

	Tmin (°C)			Tmax (°C)			Precipitation (mm)			Cholera		
	Min	Max	Mean	Min	Max	Mean	Min	Max	Mean	Min	Max	Mean
**1998**	-7 (Feb)	24.4 (Jun)	10.6	4 (Jan)	43.5 (Aug)	26.8	0	18 (Mar)	0.39	0	21 (Aug)	2.29
**1999**	-10 (Jan)	26 (Jul)	10.7	4 (Dec)	44 (Jul)	27.04	0	35 (Nov)	0.45	0	1 (Jul/Aug/Sep)	0.05
**2000**	-8 (Jan)	28 (Jul)	10.55	6 (Jan)	44 (Aug)	26.5	0	25 (Jan)	0.48	0	1 (Jul/Aug/Sep)	0.02
**2001**	-10 (Jan)	30 (Jul)	11.04	1 (Jan)	44.5 (Jul)	27.2	0	18.4 (Jan)	0.41	0	1 (Jun/Jul/Aug/Sep)	0.06
**2002**	-7 (Dec)	27 (Jul)	11.05	4 (Feb)	44.4 (Aug)	26.8	0	26 (Apr)	0.41	0	0	0
**2003**	-6 (Dec)	29.5 (Jul)	10.95	4.5 (Jan)	46 (Jul)	26.03	0	14 (May)	0.45	0	1 (Aug)	0.01
**2004**	-6 (Jan)	29 (Jun)	11.68	5 (Dec)	42.5 (Aug)	26.3	0	22 (Jan)	0.48	0	1 (Aug)	0.002
**2005**	-7 (Feb)	31 (Jul)	11.25	1.5 (Feb)	45 (Jul)	26.2	0	25 (Mar)	0.40	0	5 (Jul/Aug)	0.45
**2006**	-6.8 (Jan)	28.5 (Jul)	11.58	4.5 (Dec)	44.6 (Jul)	26.6	0	19 (Mar)	0.43	0	1 (Jul/Aug/Sep)	0.03
**2007**	-7 (Jan)	29.5 (Jul)	11.25	4 (Jan)	43.5 (Jul)	26.04	0	33 (Mar)	0.50	0	1 (Aug)	0.005
**2008**	-23 (Jan)	30.2 (Jul)	10.64	-4.2 (Jan)	45.6 (Jul)	25.7	0	13 (Dec)	0.27	0	1 (Jul/Aug/Sep)	0.07
**2009**	-7 (Jan)	28.4 (Jul)	11.03	4 (Jan)	45.8 (Jul)	26.6	0	43.01 (Mar)	0.45	0	0	0
**2010**	-6.8 (Dec)	29.4 (Jul)	11.9	8 (Feb)	47 (Jul)	28.6	0	10 (Feb)	0.21	0	0	0
**2011**	-14.2 (Jan)	29 (Jul)	10.9	1.5 (Jan)	45.8 (Jul)	26.2	0	16.6 (Jan)	0.41	0	11 (Aug)	0.37
**2012**	-7.1 (Feb)	27.4 (Jul)	11.03	5.2 (Feb)	42.8 (Jul)	25.9	0	16 (Nov)	0.40	0	0	0
**2013**	-7.5 (Jan)	28.7 (Jul)	11.32	2.5 (Mar)	46.8 (Jul)	27.4	0	10.1 (Nov)	0.21	0	1 (Jul/Sep)	0.008
**2014**	-7.8 (Feb)	30.6 (Jul)	11.38	-1.3 (Feb)	46.6 (Jul)	27.2	0	9.5 (Dec)	0.19	0	0	0
**2015**	-5.1 (Dec)	28.1 (Jul)	11.54	5.3 (Dec)	45.9 (Jun)	27.6	0	13 (Dec)	0.27	0	1 (Jul/Aug/Sep)	0.02
**2016**	-11 (Nov)	28.3 (Jul)	11.2	3.5 (Nov)	45.2 (Jun)	27.7	0	11 (Mar)	0.27	0	0	0

**Table 2:** Correlation between the number of cholera cases and climate variables during 1998–2016.

	Tmin	Tmax	Precipitation	Month	Cholera
**Tmin**	1				
**Tmax**	0.986	1			
**Precipitation**	-0.566	-0.633	1		
**Month**	0.191	0.187	-0.194	1	
**Cholera**	0.211	0.204	-0.226	0.332	1

### The results of GT

In the first step of analysis, GT results including gamma statistics and other relevant measures were calculated for 3 inputs and one output in five time series data (NL, 7DL, 14DL, 21DL, 30DL). Furthermore, different combinations were analyzed to assess their influence on the cholera disease modeling (Table 3). As shown in the Table, the minimum value of Gama (0.123) was observed when we used 21DL dataset with just precipitation (pr) as input combination. The best input data combination has a set of low values of gradient (0.22), standard error (0.03) and V-ratio (0.37) and selected as a best lag time for simulation in the next step. Interesting findings of the present study shows that the three input variables combination has a higher gamma statistic, compared with the other combinations.

**Table 3:** Gamma test results for five time-series.

	Mask	Gamma	Gradient	Standard Error	V-Ratio	Near Neighbours
**NL**	**T**_**min**_**,T**_**max**_**,pr**	0.199	-0.065	0.018	0.796	10
	**T**_**min**_**,T**_**max**_	0.197	2.462	0.015	0.788	10
	**T**_**max**_**,pr**	0.190	-0.868	0.012	0.761	10
	**T**_**min**_**,pr**	0.176	0.274	0.007	0.706	10
	**T_min_,**	0.194	-2.371	0.014	0.780	10
	**T**_**max**_	0.192	-1.555	0.017	0.768	10
	**pr**	0.145	0.288	0.039	0.582	10
**7DL**	**T**_**min**_**,T**_**max**_**,pr**	0.204	-0.500	0.015	0.817	10
	**T**_**min**_**,T**_**max**_	0.203	-0.979	0.011	0.812	10
	**T**_**max**_**,pr**	0.187	-0.690	0.009	0.749	10
	**T**_**min**_**,pr**	0.181	-0.315	0.010	0.726	10
	**T_min_,**	0.188	6.519	0.018	0.753	10
	**T**_**max**_	0.180	14.052	0.020	0.722	10
	**pr**	0.177	-0.344	0.100	0.711	10
**14DL**	**T**_**min**_**,T**_**max**_**,pr**	0.198	-0.494	0.016	0.794	10
	**T**_**min**_**,T**_**max**_	0.198	-0.074	0.010	0.795	10
	**T**_**max**_**,pr**	0.188	-0.839	0.013	0.753	10
	**T**_**min**_**,pr**	0.178	-0.416	0.009	0.712	10
	**T_min_,**	0.182	6.193	0.018	0.729	10
	**T**_**max**_	0.180	13.595	0.019	0.721	10
	**pr**	0.187	-0.789	0.120	0.748	10
**21DL**	**T**_**min**_**,T**_**max**_**,pr**	0.200	-0.343	0.015	0.801	10
	**T**_**min**_**,T**_**max**_	0.196	5.319	0.013	0.785	10
	**T**_**max**_**,pr**	0.181	-0.643	0.011	0.726	10
	**T**_**min**_**,pr**	0.179	-0.326	0.010	0.716	10
	**T_min_,**	0.185	5.036	0.019	0.740	10
	**T**_**max**_	0.174	13.716	0.019	0.698	10
	**pr**	**0.123**	**0.223**	**0.030**	**0.374**	**10**
**30DL**	**T**_**min**_**,T**_**max**_**,pr**	0.201	-0.839	0.215	0.911	10
	**T**_**min**_**,T**_**max**_	0.198	2.462	0.306	0.687	10
	**T**_**max**_**,pr**	0.196	-0.267	0.015	0.826	10
	**T**_**min**_**,pr**	0.179	-0.494	0.012	0.616	10
	**T_min_,**	0.174	2.036	0.026	0.840	10
	**T**_**max**_	0.183	9.612	0.018	0.798	10
	**pr**	0.181	0.344	0.029	0.674	10

**Fig 4:**
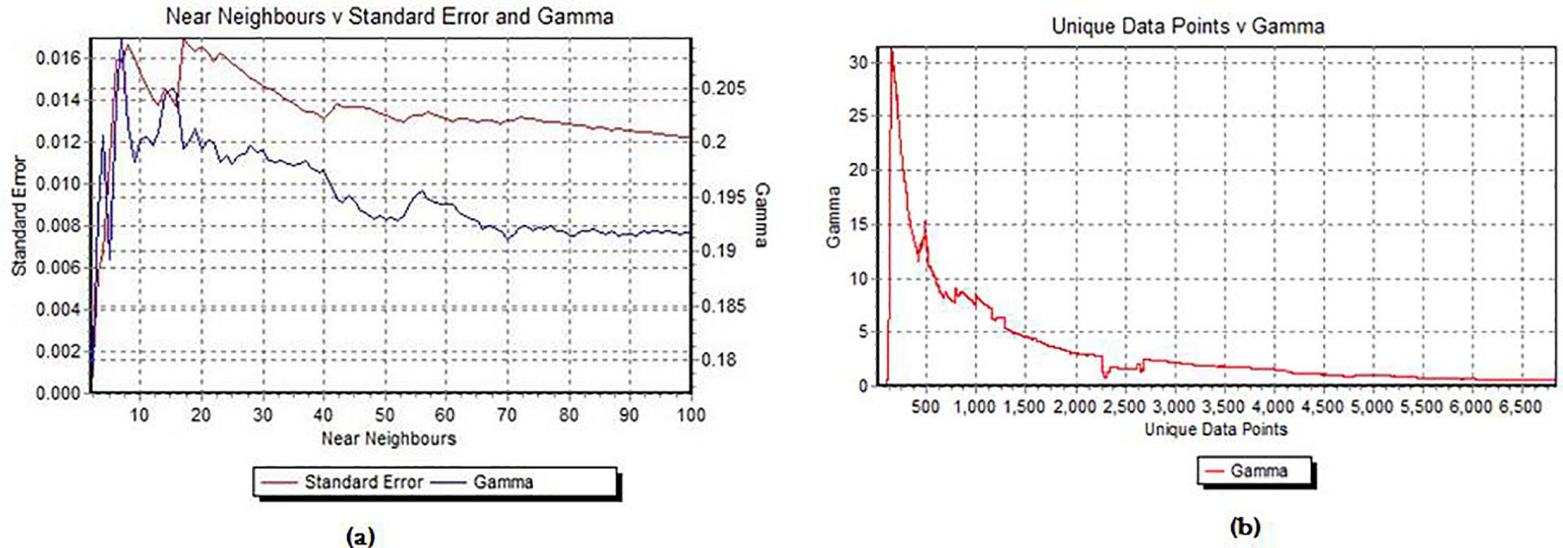
(a) Variation of gamma and SE as the number of near neighbors (b) M-test graph.

Regarding the size of dataset, the near neighbors (the number of Pmax) were estimated in 10 which shows that produces the most accurate estimate. The variation of gamma statistic and the SE versus the Pmax has been illustrated in Fig 4(A). Moreover, the sufficient data for building a smooth data model was estimated by M-test. From Fig 4(B), it can be seen that the sufficient data point to construct a smooth data model without overtraining were at least 4000–4500.

### Predictions of climate variability under future climate change scenarios

**Fig 5:**
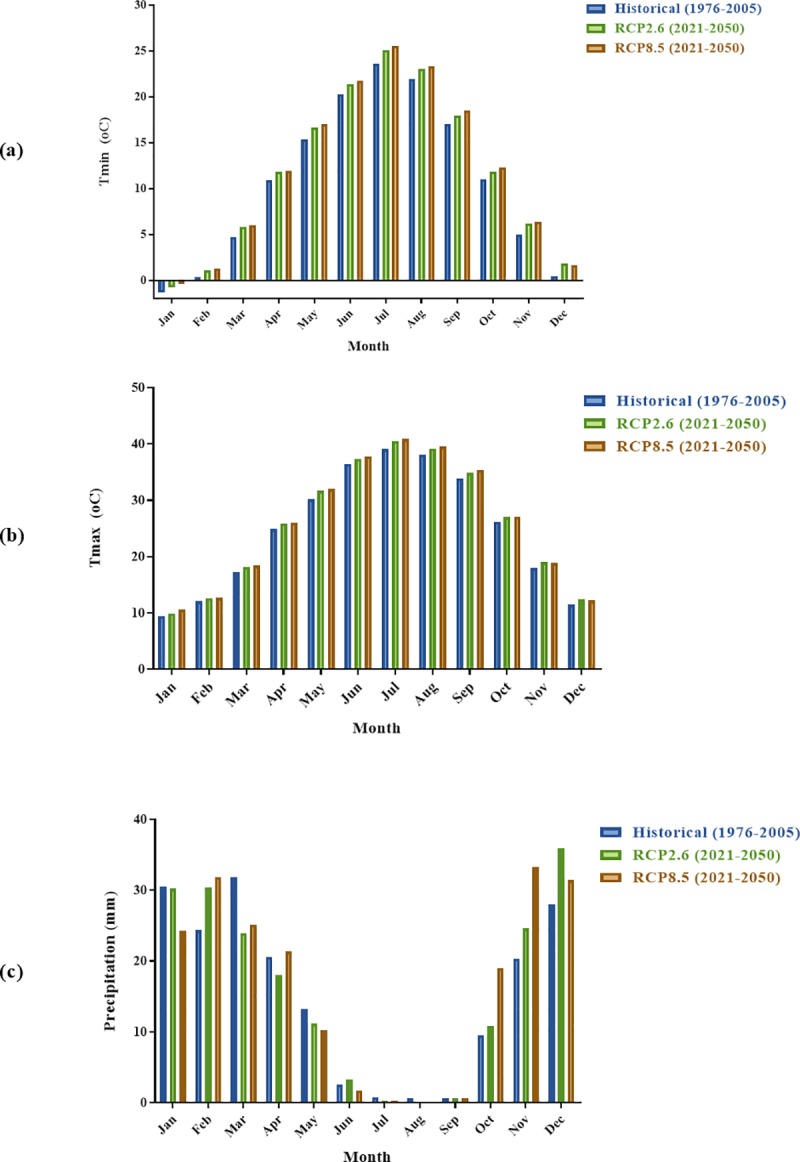
Future monthly mean of (a) minimum temperature (T_min_), (b) maximum temperature (T_max_) and (c) precipitation.

Statistical downscaling using LARS-WG was applied to define an empirical relationship between observed regional climatic (1976–2005) and GCM output models for 2021–2050 under two scenarios of RCP2.6 and RCP8.5 for the study area. Fig 5 demonstrates the downscaled monthly mean values of three parameters of precipitation, T_max_ and T_min_ under RCP2.6 and RCP8.5 scenarios, compared to the baseline values. As can be seen in this figure, RCP2.6 and RCP8.5 predicted that the minimum and maximum temperatures will rise in the study area by the year 2050. The highest increase in T_min_ and T_max_ will happen in July (1.44°C) and May (1.81°C) for RCP8.5, respectively. In addition, the lowest amount of precipitation will occur in Mar (-7.89) but in overall, RCP2.6 and RCP8.5 scenarios predicted that precipitation will decrease in the first 6 months of the year, while it will increase in the three months of winter during October, November and December.

### Cholera simulation based on ANNs

**Fig 6:**
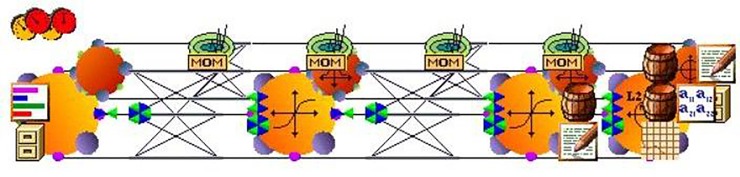
Optimized ANNs structure for simulation in the study.

The simulation of effect of climate change on cholera disease was carried out by using a feed-forward MLP type of ANNs. The schematic of the optimized NN is shown in Fig 6. This figure demonstrates that the network has one hidden layer and Tan Axon and Momentum were set as layer’s transfer function and learning rule, respectively. It is worth to mention that the results are based on data with 21-days lag (21DL). According to the results, the average final MSEs were 0.011 and 0.008 with standard deviations of 0.004 and 0.003, respectively for training and cross-validation.

**Fig 7:**
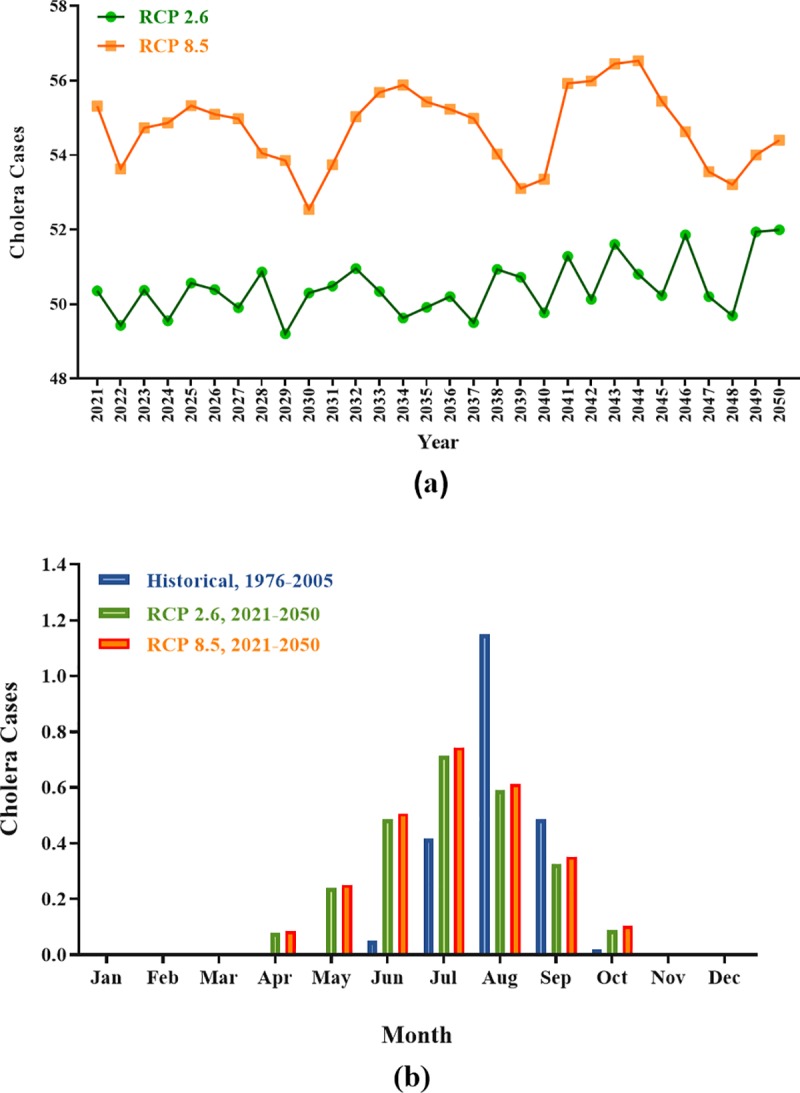
(a) Trend of cholera cases by 2050, (b) Monthly average of cholera cases.

**Fig 8:**
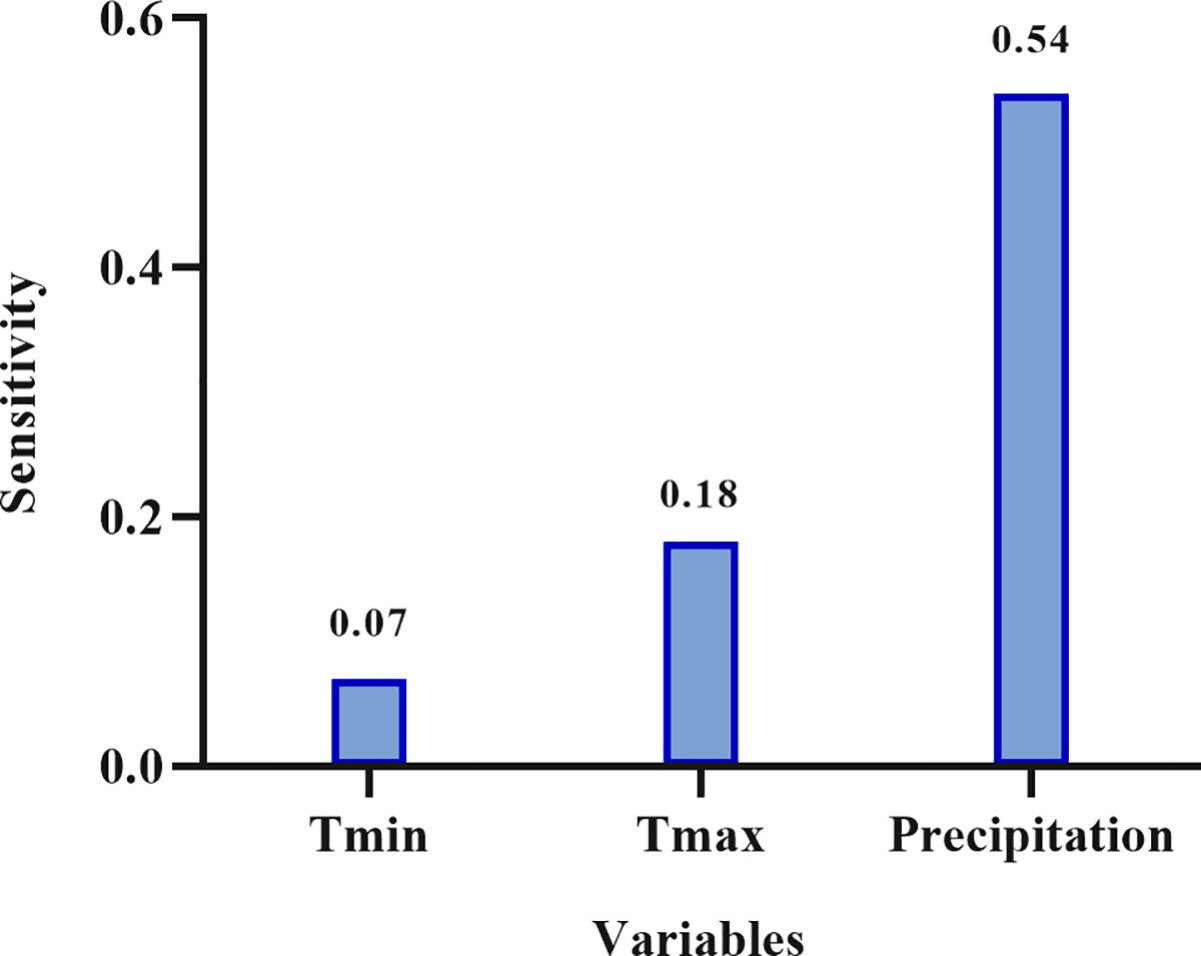
Sensitivity about the mean of each of predictors.

In evaluation of future trend of cholera disease, Fig 7(A) was prepared to show the trends of cholera cases for years of 2021–2050 under the two scenarios of RCP2.6 and RCP8.5. Also, Fig 7(B) shows monthly average of cholera cases during 2021–205. Accordingly, there is an increasing trend in cholera cases under RCP8.5 by the year 2050. The seasonal trend of cholera will change in the future qua the highest cholera cases will observe in spring and summer over next 30 years, while the highest monthly average of cholera cases in baseline period was in August. These conditions will occur when social factors such as availability of health and sanitation facilities and also safe water do not either change or become worse through the time. Modeling by ANNs and sensitivity analysis of the mean of three inputs show that precipitation had the highest effect on the incidence of cholera with sensitivity of 0.54, while T_min_ and T_max_ had sensitivity of 0.07 and 0.018, respectively (Fig 8).

## Discussion

In the present study, the effects of climate variables on cholera disease in Qom, were evaluated. Since Qom is one of the main religious tourist cities, control of cholera is a strategic and important health issue. From July 1998 and after a huge outbreak in cholera in Qom with 836 cases, the surveillance system began to identify and document all positive cases officially. The health center of Qom province assumed responsibility for finding out the source of infection, monitoring, surveillance and controlling the disease. In the 19-year time period, five waves with a 3–4 year life cycle were happened, as has been recognized in India and Bangladesh with 3-year cycles and 3–6 year cycles, respectively [36, 37]. Cholera infection can be transmitted by direct person-to-person contacts, using contaminated containers of water, unhealthy process of food preparation, or contact with contaminated environmental reservoir with persistence bacteria. However, pathways of transmission can be affected by climate conditions [38]. For example, high temperature can accelerate the growth and proliferation of pathogens in their habitats (e.g., raw food, water or environment). Rainfall can have a direct influence on transmission of cholera in two ways: first, high rainfall can increase the risk of contamination of either raw or treated water with wastewater (transmission of person-to-environment) and second, low rainfall can also increase the concentration of pathogens in water media (transmission of environment-to-person) [37]. According to the results of correlation between monthly average of climate variables and the number of cholera cases, it is obvious that high temperature with low precipitation in dry season was the ideal climatic conditions for cholera infection. In the current study, a seasonal trend was apperceived, so that the highest number of cholera cases were observed during the summer to early fall (June to October). We hypothesized that an increase in air temperatures during summer created proper environmental conditions for bacterial growth and their survival through increasing salinity. On the other hand, considerable decreasing in precipitation and increasing evaporation during dry months could lead to reducing the amount of water in surface and groundwater sources. In overall, these changes in climatic conditions could lead to increasing salinity, organic matter and aqueous temperatures and subsequent growth and proliferation of pathogens, decreasing raw water quality and increasing bacterial concentration in open water wells and the other water resources. In this regard, using contaminated and untreated water for residential consuming or irrigating vegetation could lead to increasing the risk of cholera disease incidence in high risk people. In different recent studies, environmental variables were known as most important factors in incidence of cholera. Indeed, seasonality was introduced as a stronger parameter which leads to increasing in cholera outbreaks generally during warm months [39]. The positive correlation between monthly average of temperature and cholera infection was observed in this study, which well proved by findings from East Africa, Zambia and South China [10, 11, 40]. In the case of precipitation, other studies found that heavy rainfall as an important parameter on cholera incidence can lead to flooding and affect water quality and sanitation systems [41, 42]. Although, the effect of low precipitation in increasing the risk of cholera disease described in this study, was supported by recent findings in Bangladesh and Iran [12, 21].

In the first step of analysis, to determine the foremost lag time, the best combination of inputs and to evaluate the effects of climate variables on cholera disease modeling, GT was applied. A modeler needs much time to calibrate and test different built models for all input combinations and also, there is no idea, how many data points required for calibration. The results of GT and GA can considerably reduce the model workload and help to focus on the best selection of inputs. The GA search and optimization techniques were used to evaluate the best combination of input variables for predicting the target which was chosen by smallest the asymptotic gamma statistic (the best MSE) between 2"-1 meaningful combination for a particular output [29]. The gradient and V-ratio also indicate the model complexity and the degree of predictability of given outputs [43]. Thus, a model with low gamma and smaller gradient and V-ratio are assumed as the best scenario for modeling. We indicated the impact of climate variables on cholera disease were lagged by 21 days (21DL). Our reason for choosing different lag times for variables was evaluation of the sensitivity of the cholera disease to delay in the effect of climate events. These lag times between the changes in climatic variables and the disease emergence may acquire, due to (a) time required to grow pathogens, (b) exposure to infections, (c) incubation period, (d) disease detection and (e) report delayed. Bacterial diseases have a shorter delay time than other pathogens and react more quickly to changes in temperature and precipitation [38, 44]. For example, maximum correlation coefficients between malaria incidence and sea surface temperatures in Colombia was happened with a 6 to 8 month lag [45], while the findings of Constantin de Magny in Bangladesh were shown the short time lag (one month) between environmental variables and cholera epidemics [18].

According the results of our study, just precipitation (pr) was known as the most effective input combination which means a built model by a combination of all input variables was not suitable to estimate cholera disease. On other words, the most important weather factor was only precipitation. Wang et al., used six weather input variables to estimate the evapotranspiration and their model using the combination with four input variables (maximum temperature, relative humidity, wind speed, solar radiation). They have shown that these variables have a better performance to estimate evapotranspiration and this model with more input variables could be developed more easily with less model complexity [43]. A study has reported that three contributing weather factors including daily wind speed (W), relative humidity (RH) and daily saturation vapor pressure deficit (Ed) were the best input data combination between four weather factors based on the gamma value (0.0216) [30]. If the gamma value and other three factors (i.e., gradient, Standard error and V-ratio) have low values, it is possible to build a mathematical model with high quality.

For generation of local-scale daily climate scenarios, baseline parameters of the LARS-WG (calculated from observed weather data for the period 1976–2005) modified for 2021–2050 under two RCP scenario files predicted by selected GCM model. In other words, by using this technique, the future values of local precipitation and temperature were generated for the Qom. To calibrate WG, synthetic daily weather data were generated by the parameters file in the Qom sites and then the observed and synthetic data were compared. In the current study, 10 GCMs experimental outputs were used and compared with the 30-year recorded climatic values and also, LARS-WG model applied for statistical downscaling to analyze and project future climate pattern under two scenarios of RCP2.6 and RCP8.5. Several studies applied GCMs to estimate the changes in disease in their area [46, 47]. Furthermore, some studies were used LARS-WG to generate daily site-specific climate scenarios for the future [16, 48]. The findings of this study are in agreement with Mohammadi's findings that simulated the impact of climate change on emergency medical services clients in Tehran, Iran. In fact, they predicted a wetter and warmer climate for their study area by the year 2050 [16]. In another study, the effect of climate change in the Saguenay watershed in Canada was investigated by LARS-WG downscaling technique. Their results have shown a clear increase in monthly average of both minimum and maximum temperatures (2.5 °C between current and 2080s climate) but there was not any significant changes in the daily average of precipitation in the study area [49].

The impacts of climate change on cholera disease were simulated using a feed-forward MLP type of ANNs. To build a network, the trial-and-error approach was used and to find the optimum network, training multiple times was applied. In addition, cross-validation was carried out and performance of the network was tested to protect it against overtraining. The optimized NN has one hidden layer and Tan Axon and Momentum were set as layer’s transfer function and learning rule, respectively. Different studies applied ANNs to predict the diarrheal and cholera outbreaks in distinct areas of the world [21, 22, 50, 51]. Pezeshki et al. used a multilayer perception ANNs to create a model and predict cholera disease in Chabahar, Iran. They applied monthly average of temperature, humidity and rainfall as climatic variables and their results illustrated that cholera cases were significantly related to humidity and there was a significant relationship between the cholera incidence and lack of rainfall [21]. Considering the importance of controlling the infectious diseases, many studies were done to estimate the effect of climate changes on infectious diseases in human societies in the future and prepare helpful prediction tools [52]. Accordingly, in another study, a new BPNN Model was established to predict the number of infectious diarrheas in Shanghai of China with climatic variables as input. Moreover, the results of BPNN Model were compared with SVR, RFR and MLR models. Their results indicated that BPNN model creates the best prediction results compared to the MLR, RFR and SVR models. In addition, sensitivity analysis defined that temperature-related variables (T_max_, T_min_, and T_avg_) were the effective climatic factors on infectious diarrhea while rainfall had minimum effect [51].

The projected scenario’s datasets were utilized on the optimized NN data-driven model to evaluate the future trend of cholera cases. These datasets included daily values of cholera cases and climate variables for 30 years (2021–2050). According to the present results, a slight increase was shown in cholera cases under the two scenarios of RCP2.6 and RCP8.5. Our data identified 4–5 years cycles between 2021 and 2050 under the scenario of RCP8.5. In one important study, the ‘cyclical periodicity’ of cholera disease was demonstrated in relation to the cycles of rainfall by the director of public health in Bengal. They reported that insufficient rainfall under drought conditions could lead to epidemics of cholera [53, 54]. In fact, changing the seasonality of cholera can be due to changes in rainfall patterns over the years. It should be noted that all of the conditions will happen when the authorities do not accomplish any preventive actions and social factors (availability of healthy water especially in suburb and development of wastewater systems) remain either the same or worse during the time. Considering extensive changes in global and local climate conditions in last decades and its impact on the spread of different diseases, it is a great necessity to find proper algorithms to predict the vastness of these diseases based on climate conditions. In this regards, ANNs are known as one of the appropriate tools for climate modeling and weather prediction [21]. In ANNs models, the sensitivity analysis was carried out for enhancing network performance and determination of the relative importance and contribution of input parameters on the output [55].

According to investigation of Qom Health Center, the main cause of cholera in Qom was determined as consumption of contaminated vegetables and untreated water in suburb. The justification of this phenomena can be found in the summer time. As mentioned, climate in Qom is dry and warm; therefore, lack of precipitation, drought and water shortage in the summer led to increasing bacterial load and contaminate groundwater by waterborne infection, *V*. *cholerae*. Water scarcity, made people to use untreated water (with a higher risk of contamination) from wells with lower depths for drinking and other usages in suburb. Irrigation of agricultural products by contaminated water and untreated wastewater and their consumption can transmit *V*. *cholerae* to high risk people. *V*. *cholerae* is the major cause of human diarrheal infection of cholera, which cause economic loss and mortality in developing countries. It is nominated as "the disease of poverty" because it has shown in areas with no access to adequate water treatment systems and sewage collection lines [56]. The best environmental conditions for *V*. *cholerae* are 30°C water temperature, 15% salinity, and almost alkaline environments (pH = 8.5) [57]. Human movement also is another factor which play a noticeable role in the epidemiology and transmission of infectious diseases. As mentioned, Qom is a religious city and hosts lots of tourists from endemic countries such as Iraq, Afghanistan and Pakistan annually. All of these suitable conditions were provided in Qom and it could be resulted intense growth of bacteria and increase incidence rate of cholera infection. Therefore, the promotion of any control measures such as the development of sewage collection lines, the use of treated wastewater for irrigation of farms, monitoring on the production/distribution of foods and raw vegetables can help to reduce the predicted number of cholera incidence by the model in the future.

One limitation of the present study was lack of long-term monitoring data on cholera and diarrhea disease especially in details of patient data such as age and sex categories and social and economic classes. Another limitation of our study was lack of hygienic, social and demographic information in the modeling process. Addition of some determinants affecting cholera such as availability to safe water, wastewater collection system, sanitation access, consumption of raw sewage in irrigation, hygiene in restaurants and other food providers and etc. can promote the potential of model to predict incidence of cholera with more accuracy. However, it may also require more effort, cost, and time. For further work, these impact factors can add to model and it may be helpful to use this approach to other waterborne infectious diseases.

## Conclusion

Cholera outbreaks and diarrhea disease can be linked to climatic variables and climate change. After establishing a surveillance system and recording cholera data in Qom, the information showed that the first cholera epidemic started in July 1998 and continued over the 15 years. To project the future climate using outputs from GCMs under two scenarios of the RCP2.6 and RCP8.5, we used statistical downscaling by LARS-WG as an efficient tool. Results of this study demonstrated a warmer and wetter climate pattern in our studied area by 2050. Based on the feedforward MLP type ANN technique, this study indicated that annual cholera cases will increase under both RCP scenarios during 2021–2050.

Our results showed that ANNs is a very useful and executive tool for simulation of infectious diseases such as cholera to perform preventive actions and decrease fatalities and also limit unwanted social results. But, because of some mentioned limitations of our dataset, sensitivity of simulation is not high enough. Therefore, it is important to get comprehensive and influential data including hygienic, social and demographic parameters. These results were obtained based on the present social conditions and surveillance systems. Promotion in surveillance system, increasing in the number of epidemiologic studies and identification of climatic and social risks factors can help to enhance control and prevention interventions.
